# Profile of care for children and adolescents at a gender-affirming outpatient clinic within the Brazilian National Health System in São Paulo, Brazil, between 2009 and 2020

**DOI:** 10.1590/S2237-96222024v33e2024310.especial.en

**Published:** 2025-01-10

**Authors:** Felipe Assunção de Freitas, Josevan de Souza-Silva, Bianca Machado Borba Soll, Ana Carolina Novo, Maira Caricari Saavedra, Tainá Lacroix, Alexandre Saadeh

**Affiliations:** 1 Universidade de São Paulo, Faculdade de Medicina, São Paulo, SP, Brazil; 2 Universidade de São Paulo, Faculdade de Medicina, São Paulo, SP, Brazil

**Keywords:** Personas Transgénero, Identidades de Género, Atención a la Salud de Niños y Adolescentes, Perfil Epidemiológico., Transgender People, Gender Identity, Child and Adolescent Healthcare, Epidemiological Profile

## Abstract

**Objective:**

To describe the profile of transgender children and adolescents receiving care at a gender affirming outpatient clinic within the Brazilian National Health System (*Sistema Único de Saúde* - SUS) in São Paulo.

**Methods:**

This was a descriptive study involving 555 participants, who received care between 2009 and 2020, in tertiary care services of the SUS. Data were analyzed using Pearson’s chi-square test and standard deviation, considering gender, age, and origin.

**Results:**

Between 2009 and 2020, there was an increase among males (2009, 0%; 2020, 83.3%) and a decrease among females (2009, 100%; 2020, 16.6%), with an average age of 13.7 and 14.3 years. The majority (93.7%) of consultations was performed in the Southeast region.

**Conclusion:**

Over the two decades analyzed, there was an increase in healthcare demand from male transgender children and adolescents, while there was a proportional reduction among females. Care for children and adolescents was concentrated in the Southeast region.

## Study contributions

**Main results:**

There was an increase in demand for healthcare for transgender children and adolescents, especially for those with a male gender identity. Most of the services were provided in the Southeast region.

**Implications for services:**

Healthcare services need to be equipped to accommodate this population, which has increased in recent years, especially in states outside the Southeast region. Specific needs of people with a male gender identity should be considered.

**Perspectives:**

It is essential to monitor how this phenomenon continues to evolve and to expand the range of variables under observation to better address the population’s needs. The healthcare network should be expanded to ensure universality of access.

## RESUMEN

**Objetivo:**

Describir el perfil de niños y adolescentes transgénero atendidos en una clínica ambulatoria de afirmación de género en el Sistema Único de Salud (SUS) en São Paulo.

**Métodos:**

Se realizó un estudio descriptivo con 555 participantes que recibieron atención entre 2009 y 2020 en el nivel de atención terciaria del SUS. Los datos se analizaron utilizando la prueba de chi-cuadrado de Pearson y la desviación estándar, considerando el género, la edad y el origen geográfico.

**Resultados:**

Entre 2009 y 2020, se observó un aumento en el género masculino (2009: 0%; 2020: 83,3%) y una disminución en el género femenino (2009: 100%; 2020: 16,6%), con una edad promedio de 13,7 y 14,3 años, respectivamente. La mayoría de las consultas (93,7%) se realizaron en la región Sudeste.

**Conclusión:**

En las dos décadas analizadas, hubo un aumento en la demanda de atención de niños y adolescentes transgénero del género masculino, y una reducción proporcional en el género femenino. Las consultas para niños y adolescentes trans se concentraron predominantemente en la región Sudeste.

## INTRODUCTION

Transgenderism is a phenomenon that, despite the controversies involving political and theoretical frameworks, has been present throughout the history of civilizations.^1^ Understood as an umbrella term, transgender people encompass a variety of self-declared identities under a common phenomenon: the person whose gender identity does not align with their sex assigned at birth, based on external genitalia.^1,2^

Studies on transgenderism have focused primarily on adults.^3^ Few studies have observed children and adolescents, especially in Brazil, supporting the recent focus on this topic.^2^ Interest in gender variability in childhood longstanding, with descriptions dating back to publications from the 1970s,^4^ although it was often approached as developmental delay or a psychiatric disorder.^5^

Given the tendency of the scientific and political communities to discuss health issues of the transgender population, in 2013 the Ministry of Health expanded the care guidelines previously proposed.^6^ Although it established guidelines for the adult population, it did not address the specific needs of children and adolescents.

In 2019, the Federal Council of Medicine regulated medical practice aimed at gender affirming interventions for this group, especially regarding somatic interventions, when required. The Council stipulated that puberty blockers should be administered exclusively in an experimental setting, linked to research projects submitted to ethics committees within services provided by the Brazilian National Health System (*Sistema Único de Saúde* - SUS). It also established that, for cross-sex hormone therapy, the minimum age is 16 years with parental consent, not necessarily within research protocols.^7^

There are currently two health diagnoses related to gender: gender incongruence^8^ and gender dysphoria.^9^ In order to access services within the SUS, the health concern must be classified under the International Statistical Classification of Diseases and Related Health Problems, one of the main epidemiological tools in health, designed to standardize and guide healthcare provision.

The care provided to children and adolescents seeking assistance for gender-related issues in the SUS is guided by the resolutions of the Ministry of Health and the Federal Council of Medicine. It is also guided by Resolution No. 1/2018 of the Federal Council of Psychology,^10^ which prohibits discriminatory treatments and conversion therapies in people with gender variability.

The Transdisciplinary Outpatient Clinic for Gender Identity and Sexual Orientation (*Ambulatório Transdisciplinar de Identidade de Gênero e Orientação Sexual* - AMTIGOS), of the Instituto de Psiquiatria do Hospital das Clínicas da Faculdade de Medicina da Universidade de São Paulo, offers multidisciplinary health care to those experiencing gender variability and their specific needs. An example of such needs is the elevated levels of mental distress, compared to the general population, experienced by transgender children and adolescents.^11^ This distress may arise from the emotional conflict of living in a body perceived as incongruent with their gender identity, as well as from stigma and prejudice.^12^

In Brazil, few services met the requirements for providing care to children and adolescents with gender incongruence, including AMTIGOS and the Transdisciplinary Gender Identity Program, at the Hospital de Clínicas de Porto Alegre, located in the Southeast and South regions of the country. This study aims to describe the number and distribution of children and adolescents who received care at AMTIGOS between 2009 and 2020, as well as the age profile, gender identity and geographic origin of the sample.

## METHODS

### Study design

This was a descriptive study based on data collected from the AMTIGOS service, between January 2009 and March 2020. The initial date marks the year when the first individual under the age of 18 accessed this healthcare service. The final date corresponds to the suspension of services due to the COVID-19 pandemic.

Children and adolescents aged between 3 and 18 years who accessed AMTIGOS during the specified period and who reported experiencing gender variability that met the diagnostic criteria for gender identity disorder (code F64), as proposed by the 10th edition of the World Health Organization’s International Classification of Diseases, were eligible for the study. The exclusion criteria were: included: lack of legal guardian consent for participation in the study and failure to meet the diagnostic inclusion criteria.

### Setting

The study was conducted at AMTIGOS, an institution that has been providing longitudinal care for children and adolescents with gender incongruence since 2009. The outpatient clinic consisted of a multidisciplinary team (psychiatry, psychology, pediatrics, speech therapy, nursing, and physical education). Individuals can access care through a self-referral process, initiated by email from families, non-governmental organizations, or healthcare professionals.

### Data source and variables

Data were collected through a clinical form developed by a psychiatrist. These records were submitted to the administrative department in individual files, and subsequently entered into a Microsoft Excel 365 spreadsheet. This is an anonymous database, accessible in accordance with the Access to Information Law^13^ by contacting the health institution via email: amtigos.ipq@hc.fm.usp.br.

### Study variables

The geographic regions of Brazil (North, Northeast, Midwest, Southeast and South) and states, and gender identity self-reported by children and adolescents (female, male) were included as variables.

### Statistical analysis

Data were compiled into an Excel spreadsheet and descriptively analyzed using the SPSS program version 23. Descriptive analyses included calculations of the mean, standard deviation, percentage, and the distribution of absolute numbers over time and their variations.

### Ethical aspects

The research was approved by the Research Ethics Committee of the Hospital das Clínicas da Faculdade de Medicina da Universidade of São Paulo through opinion No. 5,309,326, on 03/24/2022, certificate of submission for ethical appraisal 50280121.9.0000.0068.

## RESULTS

Between November 2009 and March 2022, a total of 555 people under 18 years of age received care by the AMTIGOS team, distributed over the years (Table 1). The number of individuals assigned male at birth who identified as female was 297 (53.5%). Those assigned female at birth who identified as male totaled 258 (46.5%).

**Table 1:** Gender identity of transgender children and adolescents according to the year of care, Brazil, 2009-2020 (n=555)

Year	Female (%)	Male (%)
2020	1 (16.6)	5 (83.3)
2019	39 (38.2)	63 (61.7)
2018	65 (45.1)	79 (54.8)
2017	46 (58.2)	33 (41.8)
2016	38 (51.3)	36 (48.7)
2015	31 (64.6)	17 (34.4)
2014	29 (72.5)	11 (27.5)
2013	21 (70.0)	9 (30.0)
2012	14 (87.5)	2 (12.5)
2011	8 (72.7)	3 (27.3)
2010	4 (100.0)	0 (0.0)
2009	1 (100.0)	0 (0.0)
Total	297 (53.5)	258 (46.5)

Regarding the percentage of female and male groups, an inversion was observed over time: until 2017, most consultations per year were for females, but from 2018 onward, consultations for males became more frequent (Table 1).

People who received care were aged 3 to 18 years, with an overall average age of 14.2±6.9 years (Table 2); the average age of individuals identifying as female was 13.7±7.2 years (initial average age = 18.0 ±0.0; final average age = 15.0 ±0) ; while for those identifying as male, the average age was 14.3±6.6 (initial average age = 15.3 ±2.8; final average age = 11.0 ±3.5) ; This decreasing trend in age over the years suggests that the service reached progressively younger individuals (Table 2).

**Table 2:** Mean age and standard deviation of transgender children and adolescents according to gender identity and year of care, Brazil, 2009-2020 (n=555)

Year	Female	Male	Total
2020	15.0 ±0	11.0 ±3.5	13.0 ±1.7
2019	9.9 ±9.2	12.2 ±9.2	11.1 ±9.2
2018	12.9 ±9.2	14.3 ±9.9	13.6 ±9.5
2017	11.8 ±9.9	14.7 ±9.2	13.2 ±9.5
2016	11.2 ±10.6	13.7 ±8.5	12.5 ±9.5
2015	10.9 ±10.6	15.9 ±3.5	13.4 ±7.1
2014	13.4 ±9.9	16.2 ±3.5	14.8 ±6.7
2013	14.8 ±9.9	14.2 ±9.9	14.5 ±9.8
2012	15.1 ±8.5	16.0 ±1.4	15.5 ±4.9
2011	14.1 ±9.9	15.3 ±2.8	14.7 ±6.4
2010	17.2 ±1.4	-	17.2 ±1.4
2009	18.0 ±0.0	-	18.0 ±0
Total	13.7 ±7.2	14.3 ±6.6	14.2 ±6.9

Regarding place of residence, the majority were from states in the Southeast region (n=509), followed by the Midwest (n=11), Northeast (n=8), South (n=7) and North (n=5) regions (Table 3).

**Table 3:** Distribution of transgender children and adolescents according to gender identity and state of care, Brazil, 2009-2020 (n=545^a^)

States	Female (%)	Male (%)	Total (%)
Midwest region	9 (0.17)	2 (0.4)	11 (2.1)
Federal District	5 (0.9)	2 (0.4)	7 (1.3)
Goiás	2 (0.4)	-	2 (0.4)
Mato Grosso do Sul	1 (0.2)	-	1 (0.2)
Mato Grosso	1 (0.2)	-	1 (0.2)
Northeast region	6 (0.12)	2 (0.4)	8 (0.16)
Bahia	2 (0.4)	1 (0.2)	3 (0.6)
Ceará	2 (0.4)	-	2 (0.4)
Piauí	1 (0.2)	-	1 (0.2)
Rio Grande do Norte	-	1 (0.2)	1 (0.2)
Sergipe	1 (0.2)	-	1 (0.2)
North region	3 (0.6)	2 (0.4)	5 (1.0)
Amazonas	1 (0.2)	1 (0.2)	2 (0.4)
Amapá	1 (0.2)	-	1 (0.2)
Rondônia	1 (0.2)	1 (0.2)	2 (0.4)
Southeast region	285 (52.3)	224 (40.4)	509 (92.6)
Minas Gerais	5 (0.9)	7 (1.3)	12 (2.2)
Rio de Janeiro	7 (1.3)	6 (1.1)	13 (2.3)
São Paulo	278 (50.1)	211 (38.0)	489 (88.1)
South region	6 (1.1)	1 (0.2)	7 (1.3)
Paraná	6 (1.1)	-	6 (1.1)
Santa Catarina	-	1 (0.2)	1 (0.2)
Total	314 (56.6)	231 (41.6)	545 (98.2)

a) 10 participants did not report their place of residence.

## DISCUSSION

In this study, an increase in the number of consultations performed per year, a reduction in the average age of individuals receiving care and an increase in the number of male-identified individuals were observed. This finding is potentially due to the population’s early access to health services.

This study has limitations in considering identities from a binary perspective, excluding those that do not fit within this framework. Qualitative studies, aiming to understand lived experiences through discourse and personal narratives, would be relevant. Alongside the increase in the number of consultations with transgender children and adolescents, a gender proportion reversal was observed, similar to previous studies, where from the 2010s onward, male-identified individuals and their guardians increasingly sought gender-affirming healthcare services earlier in life.^15-18^

Access to information and efforts to combat prejudice may be responsible for the growing number of guardians seeking guidance regarding gender variability, including during childhood and adolescence. The increasing scientific discussion on transgender and transvestite issues in Brazil is evident through the growing number of academic works, such as dissertations and theses, particularly after 2010, the year the National Policy for Comprehensive Health of LGBT was published.^16^

The increase in demand, especially among younger people, may also be related to greater knowledge, dissemination and desire for somatic interventions. With the possibility of opting to puberty blockers to prevent the development of unwanted secondary sexual characteristics, it is expected that the demand for care among prepubescent individuals, typically aged 8 to 14 years, will increase before or during the early stages of puberty.^15,17,19^

Female-assigned individuals who identify as male generally enter puberty earlier than male-assigned individuals who identify as female.^20^ Therefore, the demand for somatic interventions tends to arise at younger ages,^15,17^ ^20^ driving greater demand for healthcare services to manage somatic experiences that are inconsistent with the individual’s gender identity.

Another factor that may contribute to the increased demand among male-identified individuals is that adolescents with gender incongruence who identified as female reported higher rates of bullying.^20^ This could hinder the expression of female-identified individuals and limit spontaneous healthcare-seeking behaviors in this age group, as they may perceive it as easier for female individuals to express male identities than the oposite.^15^

The SUS must prepare to provide comprehensive care to this population, with a focus on equity. Health care for transgender children and adolescents is available in major centers in Brazil, concentrated in the Southeast region, which restricts access. Integration among teaching, service, research and community must occur through the democratic and participatory development of public policies that strengthen the educational, practical and ethical framework of healthcare students and professionals.
