# Supplementary material for: Profile of care for children and adolescents at a gender-affirming outpatient clinic within the Brazilian National Health System in São Paulo, Brazil, between 2009 and 2020
Source: Epidemiol Serv Saude. 2025 Jan 10;33(spe1):e2024310. doi: 10.1590/S2237-96222024v33e2024310.especial.en (PMC11734659; doi:10.1590/S2237-96222024v33e2024310.especial.en)
Supplement: Translation

# Translation: Perfil de atendimentos de crianças e adolescentes de um ambulatório de afirmação de gênero no Sistema Único de Saúde em São Paulo entre 2009 e 2020

**DOI:** 10.1590/S2237-96222024v33e2024310.especial.pt

**Authors:** Felipe Assunção de Freitas, Josevan de Souza-Silva, Bianca Machado Borba Soll, Ana Carolina Novo, Maira Caricari Saavedra, Tainá Lacroix, Alexandre Saadeh

**Affiliations:** 1 Universidade de São Paulo, Faculdade de Medicina, São Paulo, SP, Brasil; 2 Universidade de São Paulo, Faculdade de Medicina, São Paulo, SP, Brasil

**Keywords:** Pessoas Transgênero, Identidades de Gênero, Assistência à Saúde da Criança e do Adolescente, Perfil Epidemiológico

## Abstract

**Objetivo:**

Descrever o perfil de crianças e adolescentes transgêneros atendidos em um ambulatório de afirmação de gênero no Sistema Único de Saúde (SUS) em São Paulo.

**Métodos:**

Utilizou-se estudo descritivo, com 555 participantes, que foram atendidos entre 2009 e 2020, na atenção terciária do SUS. Os dados foram analisados por teste qui-quadrado de Pearson e desvio-padrão, considerando gênero, idade e procedência.

**Resultados:**

Entre 2009 e 2020, ocorreu aumento no gênero masculino (2009, 0%; 2020, 83,3%) e a diminuição no gênero feminino (2009, 100%; 2020, 16,6%), com média de idade de 13,7 e 14,3 anos. A maioria (93,7%) dos atendimentos foi realizada na região Sudeste.

**Conclusão:**

Nas duas décadas analisadas, houve aumento na procura por atendimento por crianças de adolescentes trans do gênero masculino, e proporcionalmente, redução no gênero feminino. Os atendimentos de crianças e adolescentes concentraram-se na região Sudeste.

## Contribuições do estudo

**Principais resultados:**

Observou-se aumento na procura do serviço de saúde para cuidados de crianças e adolescentes transgênero, principalmente para aqueles com identidade de gênero masculina. A maioria dos atendimentos ocorreu na região Sudeste.

**Implicações para os serviços:**

Os serviços devem se instrumentalizar para receber tal população que aumentou nos últimos anos, em especial nos estados fora da região Sudeste. Especificidades de pessoas com identidade de gênero masculina devem ser observadas.

**Perspectivas:**

Observar como o fenômeno continua se apresentando e ampliar o escopo de variáveis observadas, com objetivo de se aproximar das demandas da população. A ampliação de rede de serviços deve aumentar visando à universalidade.

## INTRODUÇÃO

A transgeneridade é um fenômeno que, apesar das controvérsias envolvendo referenciais políticos e teóricos, está presente na biografia das civilizações.^1^ Compreendido como termo guarda-chuva, a pessoa transgênero agrupa diferentes possibilidades de identidades autodeclaradas sob um fenômeno comum: a falta de identificação da pessoa com o gênero que lhe é atribuído, partindo da genitália externa.^1,2^

Estudos se ocuparam com a transgeneridade, sobretudo em adultos.^3^ São poucos aqueles que observaram crianças e adolescentes, especialmente no Brasil, subsidiando o recente enfoque no tema.^2^ O interesse pela variabilidade de gênero na infância é antigo, pois sua descrição parte de publicações por volta de 1970,^4^ ainda que abordado como atraso do desenvolvimento ou transtorno psiquiátrico.^5^

Diante da tendência das comunidades científica e política em discutir questões de saúde da população transgênero, em 2013 o Ministério da Saúde ampliou as linhas de cuidado propostas anteriormente.^6^ Embora tenha estabelecido norteadores para a população adulta, não contemplou as especificidades das crianças e dos adolescentes

Em 2019, o Conselho Federal de Medicina regulamentou a prática médica destinada às intervenções de afirmação de gênero nesse grupo, em especial quanto às intervenções somáticas, quando demandadas. Esse Conselho determinou que o bloqueio puberal deve ocorrer exclusivamente em caráter experimental e vinculado a projetos de pesquisa submetidos ao sistema de comitês de ética em pesquisa, em serviços do Sistema Único de Saúde (SUS). Estabeleceu também que, para a terapia hormonal cruzada, a idade mínima é de 16 anos com autorização dos responsáveis, não necessariamente em protocolos de pesquisa.^7^

Existem dois diagnósticos em saúde vigentes em relação ao gênero: incongruência degênero^8^ e disforia de gênero.^9^ No SUS, para se ter acesso aos serviços, é necessário que a demanda em saúde esteja categorizada na Classificação Estatística Internacional de Doenças e de Problemas Relacionados à Saúde, uma das principais ferramentas epidemiológicas em saúde que tem, entre os objetivos, padronizar e orientar a assistência.

O atendimento de crianças e adolescentes que procuram assistência devido a questões de gênero no SUS é orientado pelas resoluções do Ministério da Saúde e do Conselho Federal de Medicina. O atendimento também é orientado pela Resolução nº 1/2018 do Conselho Federal de Psicologia,^10^ que veta tratamentos discriminatórios e terapias conversivas em pessoas com variabilidade de gênero. 

O Ambulatório Transdisciplinar de Identidade de Gênero e Orientação Sexual (AMTIGOS), do Instituto de Psiquiatria do Hospital das Clínicas da Faculdade de Medicina da Universidade de São Paulo, oferece acompanhamento multiprofissional em saúde àqueles que vivenciam variabilidade de gênero em suas demandas e particularidades. Um exemplo de tais particularidades é o fato de que crianças e adolescentes com variabilidade de gênero apresentam níveis elevados, quando comparados à população geral, de situações de sofrimento mental.^11^ Esses problemas podem ser gerados tanto pelo conflito emocional de lidar com um corpo entendido como incongruente à identidade de gênero, quanto pelo estigma epreconceito.^12^

No Brasil, poucos serviços contemplavam os requisitos para a realização de atendimentos de crianças e adolescentes com incongruência de gênero, entre eles o AMTIGOS e o Programa Transdisciplinar de Identidade de Gênero, do Hospital de Clínicas de Porto Alegre, localizados no Sudeste e no Sul do país. Este trabalho objetiva descrever o número e a distribuição de crianças e adolescentes que passaram por atendimentos no AMTIGOS entre 2009 e 2020, assim como o perfil etário, a identidade de gênero e a procedência geográfica da amostra.

## MÉTODOS

### Delineamento

Trata-se de estudo descritivo dos dados produzidos no atendimento do AMTIGOS, realizado entre janeiro de 2009 e março de 2020. Tal período foi marcado inicialmente no ano em que a primeira pessoa menor de 18 anos ingressou no equipamento de saúde. A data final referiu-se ao momento em que os atendimentos foram paralisados em decorrência da pandemia de covid-19. 

Foram elegíveis crianças e adolescentes entre 3 e 18 anos de idade que acessaram o AMTIGOS no período descrito e que apresentavam relatos de vivência de variabilidade de gênero que contemplavam os critérios diagnósticos para transtorno de identidade sexual (categoria F64), conforme proposto pela 10ª edição do Código Internacional de Doenças da Organização Mundial de Saúde. Os critérios de exclusão foram: não ter autorização dos responsáveis legais para participação no estudo e não ter o diagnóstico relatado no critério de inclusão.

### Contexto

O estudo foi realizado no AMTIGOS, instituição que acompanha desde 2009 crianças e adolescentes com incongruência de gênero de forma longitudinal. O ambulatório era formado por uma equipe multidisciplinar (psiquiatria, psicologia, pediatria, fonoaudiologia, enfermagem e educação física). As pessoas podem ser inseridas no cuidado, por meio de fluxo de atendimento de livre demanda, sendo encaminhadas por e-mail pelas próprias famílias, por organizações não governamentais e por profissionais de saúde.

### Fonte de dados e variáveis

Os dados foram coletados por meio da ficha de atendimento elaborada por um psiquiatra. Esses dados foram encaminhados para o setor administrativo, em fichas individuais, nas quais foram registrados em planilha de Microsoft Excel 365. Trata-se de base de dados anônima, acessível de acordo com a Lei de Acesso à Informação^13^ por meio de solicitação à instituição de saúde pelo e-mail: amtigos.ipq@hc.fm.usp.br.

### Variáveis do estudo

Foram incluídas como variáveis as regiões geográficas do Brasil (Norte, Nordeste, Centro-Oeste, Sudeste e Sul) e estados, identidade de gênero autorreferida pelas crianças e dos adolescentes (feminino, masculina).

### Análise estatística

Os dados foram compilados em planilha de Excel e analisados descritivamente com auxílio do programa SPSS versão 23. Os dados foram avaliados descritivamente por meio de cálculo da média, desvio-padrão, percentual e a distribuição do número absoluto ao longo do tempo e suas variações. 

### Aspectos éticos

A pesquisa foi aprovada pelo Comitê de Ética de Pesquisa do Hospital das Clínicas da Faculdade de Medicina da Universidade de São Paulo por meio parecer nº 5.309.326 de 24/03/2022, certificado de apresentação de apreciação ética 50280121.9.0000.0068.

## RESULTADOS

Entre novembro de 2009 e março de 2022, foram realizados 555 atendimentos de pessoas menores de 18 anos pela equipe do AMTIGOS, distribuídas ao longo dos anos (Tabela 1). O número de pessoas que tiveram o sexo masculino ao nascer e se identificam com o gênero feminino foi de 297 (53,5%). Aquelas pessoas que tiveram o sexo feminino ao nascer e se identificam com o gênero masculino foi de 258 (46,5%)

**Tabela 1:** Identidade de gênero de crianças e adolescentes trans de acordo com o ano de atendimento, 2009-2020 (n=555)

Ano	Feminina (%)	Masculina (%)
2020	1 (16,6)	5 (83,3)
2019	39 (38,2)	63 (61,7)
2018	65 (45,1)	79 (54,8)
2017	46 (58,2)	33 (41,8)
2016	38 (51,3)	36 (48,7)
2015	31 (64,6)	17 (34,4)
2014	29 (72,5)	11 (27,5)
2013	21 (70,0)	9 (30,0)
2012	14 (87,5)	2 (12,5)
2011	8 (72,7)	3 (27,3)
2010	4 (100,0)	0 (0,0)
2009	1 (100,0)	0 (0,0)
Total	297 (53,5)	258 (46,5)

Em relação ao percentual dos grupos dos gêneros feminino e masculino, foi percebido que, ao longo dos anos, ocorreu uma inversão: até 2017, a maior parte dos atendimentos por ano era de pessoas do gênero feminino e, em 2018, passou a ser do gênero masculino (Tabela 1).

As pessoas que passaram por atendimento tinham entre 3 e 18 anos, com idade média geral em 14,2±6,9 anos (Tabela 2); sendo a idade média do grupo de pessoas com identidade de gênero feminino de 13,7±7,2 anos (idade média inicial=18,0±0,0; idade média final=15,0±0); e a do grupo de pessoas do gênero masculino, de 14,3±6,6 (idade média inicial=15,3±2,8; idade média final= 11,0±3,5);tal dado mostrou-se decrescente ao longo dos anos, sugerindo que o serviço atingiu cada vez mais jovens (Tabela 2).

**Tabela 2:** Média da idade e desvio padrão de crianças e adolescentes trans de acordo com a identidade de gênero e ano de atendimento, 2009-2020 (n=555)

Ano	Feminina	Masculina	Total
2020	15,0±0	11,0±3,5	13,0±1,7
2019	9,9±9,2	12,2±9,2	11,1±9,2
2018	12,9±9,2	14,3±9,9	13,6±9,5
2017	11,8±9,9	14,7±9,2	13,2±9,5
2016	11,2±10,6	13,7±8,5	12,5±9,5
2015	10,9±10,6	15,9±3,5	13,4±7,1
2014	13,4±9,9	16,2±3,5	14,8±6,7
2013	14,8±9,9	14,2±9,9	14,5±9,8
2012	15,1±8,5	16,0±1,4	15,5±4,9
2011	14,1±9,9	15,3±2,8	14,7±6,4
2010	17,2±1,4	-	17,2±1,4
2009	18,0±0,0	-	18,0±0
Total	13,7±7,2	14,3±6,6	14,2±6,9

Quanto ao local de moradia, a maioria residiam em estados do Sudeste (n= 509), seguido das regiões Centro-Oeste (n=11), Nordeste (n=8), Sul (n=7) e Norte (n=5) (Tabela 3).

**Tabela 3:** Distribuição das crianças e adolescentestrans de acordo com a identidade de gênero e estado de atendimento, 2009-2020 (n=545ª)

Estados	Feminina (%)	Masculina (%)	Total (%)
Centro-Oeste	9 (0,17)	2 (0,4)	11 (2,1)
Distrito Federal	5 (0,9)	2 (0,4)	7 (1,3)
Goiás	2 (0,4)	-	2 (0,4)
Mato Grosso do Sul	1 (0,2)	-	1 (0,2)
Mato Grosso	1 (0,2)	-	1 (0,2)
Nordeste	6 (0,12)	2 (0,4)	8 (0,16)
Bahia	2 (0,4)	1 (0,2)	3 (0,6)
Ceará	2 (0,4)	-	2 (0,4)
Piauí	1 (0,2)	-	1 (0,2)
Rio Grande do Norte	-	1 (0,2)	1 (0,2)
Sergipe	1 (0,2)	-	1 (0,2)
Norte	3 (0,6)	2 (0,4)	5 (1,0)
Amazonas	1 (0,2)	1 (0,2)	2 (0,4)
Amapá	1 (0,2)	-	1 (0,2)
Rondônia	1 (0,2)	1 (0,2)	2 (0,4)
Sudeste	285 (52,3)	224 (40,4)	509 (92,6)
Minas Gerais	5 (0,9)	7 (1,3)	12 (2,2)
Rio de Janeiro	7 (1,3)	6 (1,1)	13 (2,3)
São Paulo	278 (50,1)	211 (38,0)	489 (88,1)
Sul	6 (1,1)	1 (0,2)	7 (1,3)
Paraná	6 (1,1)	-	6 (1,1)
Santa Catarina	-	1 (0,2)	1 (0,2)
Total	314 (56,6)	231 (41,6)	545 (98,2)

a) 10 participantes não informaram residência.

## DISCUSSÃO

Neste estudo, foi observado aumento no número de atendimentos realizados por ano, a redução na idade média das pessoas atendidas e o aumento no número de pessoas com identidades masculinas. Potencialmente o achado se deve ao acesso precoce da população ao serviço de saúde.

O presente estudo tem limitações ao considerar as identidades em uma perspectiva binária, desconsiderando aquelas que não se localizem nessa estrutura. Estudos de natureza qualitativa, visando compreender as vivências por meio de discurso e da experiência das pessoas pesquisadas, seriam relevantes. Concomitantemente ao aumento do número de atendimentos realizados com crianças e adolescentes transgênero, observou-se inversão na proporção de gênero dos participantes, semelhante a pesquisas prévias, onde a partir da década de 2010, pessoas do gênero masculino e seus responsáveis procuraram os equipamentos de saúde especializados em afirmação de gênero, de forma mais proeminente e cada vez mais cedo.^15-18^

O acesso à informação e o combate ao preconceito podem ser responsáveis pela maior procura por parte dos cuidadores por orientação a respeito das vivências de variabilidade de gênero, incluindo na infância e na adolescência. Houve o aumento na discussão científica da temática travestis e transexuais no Brasil, que pode ser percebido a partir do aumento no número de trabalhos, como dissertações e teses, especialmente após 2010, ano no qual foi publicada a Política Nacional de Saúde Integral LGBT.^16^

O aumento na procura, principalmente por pessoas mais jovens, pode estar também relacionado ao maior conhecimento, à divulgação e ao desejo por intervenções somáticas. Com a possibilidade de recorrer ao bloqueio puberal, para impedir o desenvolvimento de caracteres sexuais secundários correspondentes ao sexo e indesejados, é esperado que a demanda por acompanhamento por pessoas antes da puberdade, geralmente entre 8 e 14 anos, aumente em idades anteriores a tal estágio de desenvolvimento, ou ainda diante das primeiras mudanças percebidas no início dessa fase.^15,17,19^

Pessoas do sexo feminino e que apresentam identidades de gênero masculina geralmente iniciam a puberdade em idades anteriores às pessoas pertencentes ao sexo masculino e que se identificam com o gênero feminino.^20^ Nesse sentido, é esperado que as demandas por intervenções somáticas se tornem mais salientes em idades menores^15,17-20,^,instigando a maior procura por serviços de saúde para lidar com a experiência somática fora do esperado para o gênero com o qual se identifica. 

Outro fator que pode interferir no aumento da procura de pessoas com identidades de gênero masculinas é o fato de que adolescentes com incongruência de gênero que se identificaram com identidade feminina relataram maior experiência de *bullying*.^20^ Isso dificultaria a expressão das pessoas com identidade feminina e a procura espontânea pelo serviço nessa faixa etária específica, por perceberem maior facilidade para pessoas do feminino expressarem identidades masculinas que o oposto.^15^

O SUS deve se preparar para o atendimento integral dessa população, na perspectiva da equidade. O cuidado em saúde voltado para crianças e adolescentes transgêneros se encontra em grandes centros brasileiros, concentrados na região Sudeste, o que restringe o acesso. A integração entre ensino, serviço, pesquisa e comunidade deve acontecer por meio da construção de forma democrática e participativa políticas públicas que fortaleçam o arcabouço formativo, prático e ético de estudantes e profissionais de saúde.
